# Genes Activated by *Vibrio cholerae* upon Exposure to *Caenorhabditis elegans* Reveal the Mannose-Sensitive Hemagglutinin To Be Essential for Colonization

**DOI:** 10.1128/mSphereDirect.00238-18

**Published:** 2018-05-23

**Authors:** Cornelia List, Andreas Grutsch, Claudia Radler, Fatih Cakar, Franz G. Zingl, Kristina Schild-Prüfert, Stefan Schild

**Affiliations:** a Institute of Molecular Biosciences, University of Graz, Graz, Austria; b BioTechMed-Graz, Graz, Austria; University of Kentucky; Boston Children's Hospital; Umeå University; Technische Universität Braunschweig

**Keywords:** MSHA, RIVET, bacterial grazing, bacterivorous, chitin, development, environment, growth retardation, nematodes, nutrition, predator model, survival

## Abstract

During its life cycle, the facultative human pathogen Vibrio cholerae, which is the causative agent of the diarrheal disease cholera, needs to adapt to a variety of different conditions, such as the human host or the aquatic environment. Importantly, cholera infections originate from the aquatic reservoir where V. cholerae persists between the outbreaks. In the aquatic environment, bacteria are constantly threatened by predatory protozoa and nematodes, but our knowledge of the response pathways and adaptation strategies of V. cholerae to such stressors is limited. Using a temporally controlled reporter system of transcription, we identified more than 100 genes of V. cholerae induced upon exposure to the nematode Caenorhabditis elegans, which emerged recently as a valuable model for environmental predation during the aquatic lifestyle of V. cholerae. Besides others, we identified and validated the genes encoding the mannose-sensitive hemagglutinin (MSHA) type IV pilus to be significantly induced upon exposure to the nematode. Subsequent analyses demonstrated that the mannose-sensitive hemagglutinin is crucial for attachment of V. cholerae in the pharynx of the worm and initiation of colonization, which results in growth retardation and developmental delay of C. elegans. Thus, the surface adhesion factor MSHA could be linked to a fitness advantage of V. cholerae upon contact with bacterium-grazing nematodes.

**IMPORTANCE** The waterborne diarrheal disease cholera is caused by the bacterium Vibrio cholerae. The facultative human pathogen persists as a natural inhabitant in the aquatic ecosystem between outbreaks. In contrast to the human host, V. cholerae requires a different set of genes to survive in this hostile environment. For example, predatory micrograzers are commonly found in the aquatic environment and use bacteria as a nutrient source, but knowledge of the interaction between bacterivorous grazers and V. cholerae is limited. In this study, we successfully adapted a genetic reporter technology and identified more than 100 genes activated by V. cholerae upon exposure to the bacterium-grazing nematode Caenorhabditis elegans. This screen provides a first glimpse into responses and adaptational strategies of the bacterial pathogen against such natural predators. Subsequent phenotypic characterization revealed the mannose-sensitive hemagglutinin to be crucial for colonization of the worm, which causes developmental delay and growth retardation.

## Abstract (precis)

The waterborne diarrheal disease cholera is caused by the bacterium Vibrio cholerae. The facultative human pathogen persists as a natural inhabitant in the aquatic ecosystem between outbreaks. In contrast to the human host, V. cholerae requires a different set of genes to survive in this hostile environment. For example, predatory micrograzers are commonly found in the aquatic environment and use bacteria as a nutrient source, but knowledge of the interaction between bacterivorous grazers and V. cholerae is limited. In this study, we successfully adapted a genetic reporter technology and identified more than 100 genes activated by V. cholerae upon exposure to the bacterium-grazing nematode Caenorhabditis elegans. This screen provides a first glimpse into responses and adaptational strategies of the bacterial pathogen against such natural predators. Subsequent phenotypic characterization revealed the mannose-sensitive hemagglutinin to be crucial for colonization of the worm, which causes developmental delay and growth retardation.

## INTRODUCTION

The Gram-negative bacterium Vibrio cholerae is the causative agent of the severe human diarrheal disease cholera, which is now endemic in many areas of the African and Asian continents (1). Lately, an increasing frequency of major epidemics, including, e.g., Zimbabwe (2009), Haiti (2010), and Yemen (ongoing), has drawn attention from the public. However, these local outbreaks represent just the tip of the iceberg of this devastating disease with an overall global burden estimated to be 3 to 5 million cases and up to 130,000 deaths per year (2). Particularly children show a high mortality rate in developing countries, where diarrheal diseases remain the second most common cause of death.

Hallmarks of the life cycle of the clinically relevant V. cholerae isolates are the transitions between two dissimilar habitats, i.e., as a natural inhabitant of aquatic ecosystems and as a pathogen in the human gastrointestinal tract (3, 4). The fast adaptation of V. cholerae between the alternative lifestyles is remarkable and represents a key feature for the transition fitness between the different stages of the V. cholerae life cycle, allowing rapid spread of cholera. At the beginning of an outbreak, a relatively high infection dose of 10^6^ to 10^9^ CFU is required to cause disease (5). However, after passage through the host V. cholerae exits in a transient hyperinfectious state. For reinfections of humans within this period, the infectious dose can drop down to 10^2^ to 10^3^ CFU (6, 7). As outbreaks generally originate from the aquatic reservoir, a better understanding of the whole life cycle of V. cholerae with an emphasis on the persistence and survival of V. cholerae in the aquatic ecosystems has become a major target for the development of new therapeutic strategies and the containment of the disease (1, 2, 5, 8, 9).

V. cholerae spends much of its life cycle outside the host in estuarine and costal aquatic reservoirs with a geographical range from tropics to temperate water worldwide. The capability of V. cholerae to survive in many different environmental niches requires adaptation to a number of fluctuating conditions, including temperature shifts, osmotic stress, and nutrient limitation (10). To survive these challenges, V. cholerae employs a number of strategies, including formation of biofilms on abiotic and biotic surfaces, the conversion into a viable but nonculturable (VBNC) state under unfavorable conditions, and the acquisition and storage of nutrients.

In addition, bacterivorous predators like free-living protozoa and nematodes are a constant threat for environmental bacteria (11). Vice versa, bacteria have evolved protective responses to such biological stressors. In order to study the interactions between V. cholerae and bacterium-grazing invertebrates, the nematode Caenorhabditis elegans has been successfully established as a natural predator model. So far, two secreted effectors of V. cholerae, the protease PrtV and the hemolysin HlyA, have been identified to cause lethality or developmental delay in the nematode C. elegans and thereby contribute to protection from natural predator grazing (12, 13). A microarray study compared the host gene expression response of C. elegans exposed to V. cholerae wild type (WT) and an *hlyA* mutant (14). Several genes specifically induced by the worm in response to HlyA have been identified, including pathways previously implicated in innate immune response pathways (14). Furthermore, a recent study demonstrated that the quorum-sensing molecule CAI-1 of V. cholerae is recognized by C. elegans as an attractant via the AWC^ON^ neuron (15). These studies are the first indications of defined responses by C. elegans upon contact with V. cholerae and provide a first glimpse into cross-kingdom interactions.

In contrast, transcriptional changes taking place in V. cholerae upon contact with such bacterium-grazing nematodes have not been investigated. In this study, the recombination-based *in vivo* expression technology (RIVET) was adapted to identify more than 100 V. cholerae genes activated upon exposure to the nematode, including those for the biogenesis of the mannose-sensitive hemagglutinin (MSHA). Subsequent mutagenesis and phenotypic analyses revealed MSHA to be an important factor enabling V. cholerae to colonize the pharynx of C. elegans.

## RESULTS

### Identification of genes activated upon exposure to C. elegans (*aec*).

**FIG 1:**
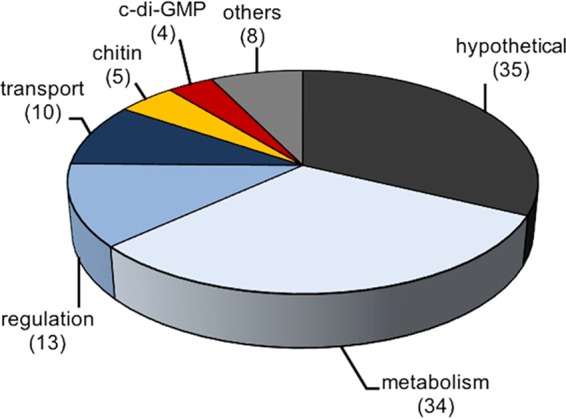
Functional distribution of genes activated upon exposure to C. elegans (*aec* genes). Shown are *aec* genes identified with recombinase-based screening technology, allocated in functional groups by their proposed function according to KEGG (http://www.genome.jp/kegg/) (43–45). The number of *aec* genes in the respective group is indicated in parentheses.

To identify genes of V. cholerae activated upon exposure to the bacterium-grazing nematode C. elegans (*aec*), we screened a library with approximately 12,000 random transcriptional gene-*tnpR* fusions, previously used to identify genes induced *in vivo* or in biofilm (16, 17). We recovered and sequenced gene-*tnpR* fusions of ~200 resolved strains, which resulted in the identification of 109 plus-stranded fusions to different genes (see Table S1 in the supplemental material). Figure 1 provides an overview of the *aec* genes divided into functional groups: 35 of the *aec* genes are hypothetical, 34 are implicated in metabolism, 13 are predicted to be involved in signaling and regulatory pathways, 10 are involved in transport, 5 have a role in chitin utilization or attachment, 4 are involved in c-di-GMP metabolism, and 8 do not fit into one of these groups and are therefore named “others.” In addition, a gene ontology analysis was performed using PANTHER (http://pantherdb.org), which supports enrichment analysis using pathway classifications from the reactome pathway database (18). Using the “biological process” classification, which groups the genes into nine subgroups (biological regulation, cellular component organization or biogenesis, cellular processes, localization, locomotion, metabolic process, multicellular organismal processes, reproduction, and response to stimulus), the categories “metabolic processes” (*P* = 0.03) and “biological regulation” (*P* = 0.01) were found to be significantly overrepresented in the identified *aec* genes with regard to their abundance in the V. cholerae genome.

10.1128/mSphereDirect.00238-18.5 TABLE S1  V. cholerae genes activated upon exposure to C. elegans. Download TABLE S1, DOCX file, 0.03 MB. Copyright © 2018 List et al. 2018 List et al. This content is distributed under the terms of the Creative Commons Attribution 4.0 International license.

**FIG 2:**
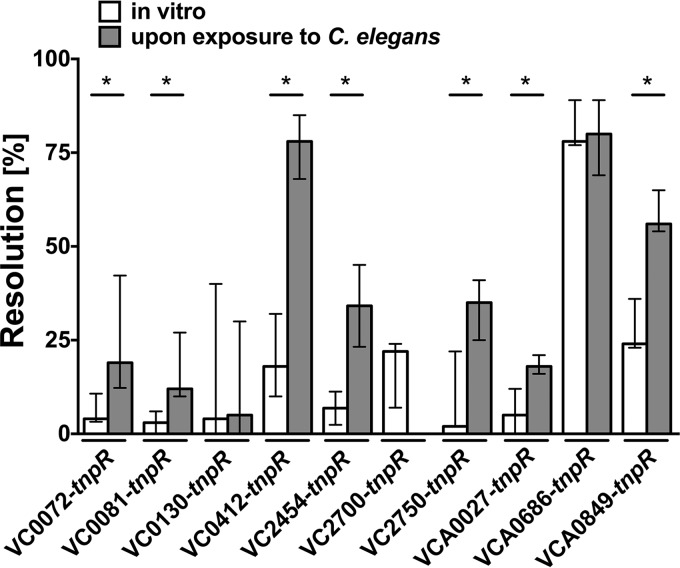
Validation of *aec* genes. Shown are the median resolution frequencies of reconstructed *tnpR*-fusion strains (indicated on the *x* axis) under *in vitro* conditions (open bars) and upon exposure to C. elegans (gray bars). The error bars indicate the interquartile ranges. Each data set consists of at least 6 independent experiments. A significant increase in the resolution frequency between *in vitro* conditions and upon exposure to C. elegans is indicated by an asterisk (*, *P* < 0.05 by Mann-Whitney U test).

Ten representative gene-*tnpR* fusions were selected for a validation process and used to reconstruct unresolved strains. Resolution frequencies of the reconstructed strains were determined under *in vitro* control conditions (growth on nematode growth medium [NGM] agar plates without C. elegans) as well as after exposure to C. elegans to determine the accuracy of the screen (see Materials and Methods for details). A significantly higher resolution frequency upon exposure to C. elegans than that for the control condition was observed for 7 out of 10 reconstructed gene-*tnpR* fusion strains (Fig. 2), which corresponds to an accuracy rate of approximately 70%. Notably, several of the identified *aec* genes are important for attachment to chitin or its utilization as a nutrient source (19). Two (VC0412 and VCA0027) could be verified to be bona fide *aec* genes in the validation process. Especially the VC0412-*tnpR* fusions demonstrated a robust increase in resolution frequency upon exposure to C. elegans over that for the control conditions. VC0412 belongs to the *msh* operon essential for synthesis of the mannose-sensitive hemagglutinin (MSHA) type IV pilus (20). To investigate the role of the MSHA pilus upon contact with C. elegans in more detail, we constructed the MSHA-negative *ΔmshA* mutant with an in-frame deletion of VC0409, encoding the major subunit of the pilus (21, 22).

### MSHA promotes attachment of V. cholerae in the pharynx of C. elegans.

**FIG 3:**
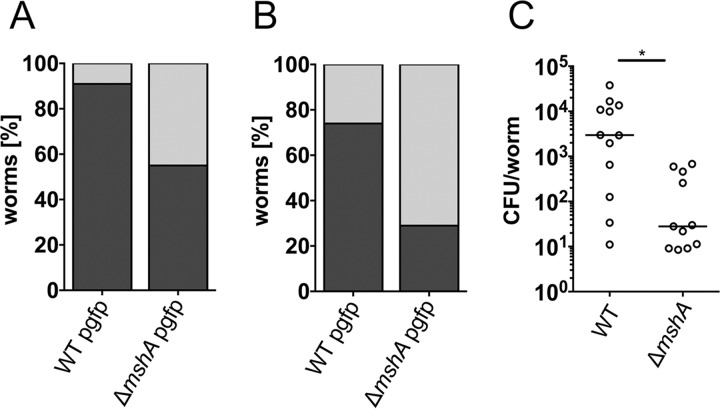
MSHA is crucial for colonization of C. elegans by V. cholerae. (A and B) Shown is the percentage of worms with detectable signal (dark gray) or no detectable signal (light gray) in the pharynx after grazing on V. cholerae WT pgfp or Δ*mshA* mutant pgfp before (A) or after (B) passage via E. coli OP50 for 2 h. At least 100 worms of each data set were analyzed. In both cases (A and B), the distributions of worms with and without signal are significantly different between nematodes grazing on V. cholerae WT pgfp and those grazing on Δ*mshA* mutant pgfp (*, *P* < 0.05 by Fisher exact test). (C) Shown are the colonization levels of worms grazing on V. cholerae WT or Δ*mshA* mutant after passage via E. coli OP50 for 2 h. Each circle represents the colonization level determined from 7 to 12 worms pooled for homogenization. The CFU were divided by the number of worms present in the respective homogenized pool to retrieve the CFU per worm (for details, see “Quantification of bacterial colonization” in Materials and Methods). The horizontal bar indicates the median of each data set. A significant difference is indicated by an asterisk (*, *P* < 0.05 by Mann-Whitney U test).

**FIG 4:**
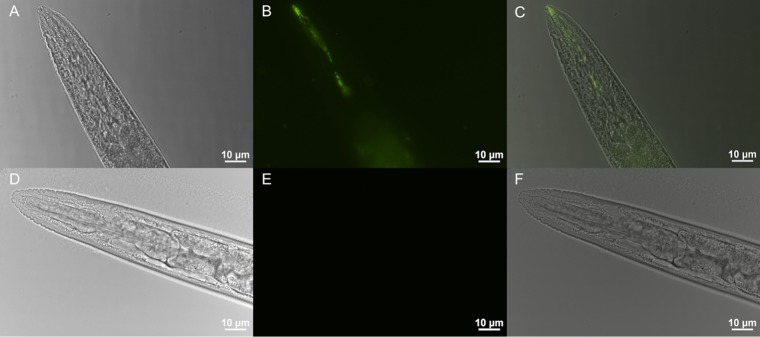
Localization of V. cholerae in the pharynx of C. elegans depends on MSHA. Representative epifluorescence microscopy images of the anterior parts, including the pharynx of worms cultivated on plates with V. cholerae WT pgfp (A to C) or V. cholerae Δ*mshA* pgfp (D to F) after a passage via E. coli OP50 for 2 h. GFP signal was detected in the green channel (excitation of 400 nm, emission of 500 to 550 nm). Shown are bright-field images (A and D), fluorescent images (B and E), and merged images (C and F).

The MSHA pilus facilitates surface adhesion of V. cholerae, which is a prerequisite for successful biofilm formation on chitinous surfaces provided by zooplankton in the aquatic environment (19). Chitin is present on nematode surfaces, and a recent study demonstrated that the polymer is an indispensable component of the chitin decoration of the C. elegans pharynx localized to specific regions of the lumen wall (23). Taking into account the impact of MSHA on development and the identification of several genes of the chitin utilization program, it could be hypothesized that V. cholerae can colonize the pharynx, with MSHA being a relevant factor for attachment. The impact of MSHA on a potential colonization of V. cholerae in the pharynx of C. elegans was investigated by fluorescence microscopy. C. elegans was cultivated on either the green fluorescent protein (GFP)-labeled V. cholerae wild type (WT) or the Δ*mshA* mutant before the nematodes were analyzed by microscopy. A comprehensive evaluation of microscopic images with more than 100 worms revealed that almost all worms grazing on V. cholerae WT pgfp (constitutive expression of GFP from a plasmid) exhibited a fluorescent signal in the pharynx, while only approximately 50% of worms grazing on Δ*mshA* pgfp showed a signal above the limit of detection (Fig. 3A). To prove that the visualized bacteria are indeed attached to the pharyngeal luminal surface, worms were passaged for another 2 h via NGM agar plates seeded with Escherichia coli OP50 without GFP expression to remove luminal or loosely attached bacteria inside the worms. After this passage, approximately 75% of the worms grazing on V. cholerae WT pgfp still showed a detectable fluorescent signal, while only in 25% of worms grazing on Δ*mshA* pgfp was a signal detected (Fig. 3B). Representative images showing the fluorescent intensity and signal localization in worms grazing on V. cholerae WT pgfp or Δ*mshA* pgfp after a 2-h passage on OP50 are provided in Fig. 4. While in worms fed on *mshA* mutants no or only low fluorescence was observed, nematodes grazing on GFP-labeled V. cholerae WT showed a reproducible signal in the pharynx. To obtain quantitative data, colonization levels were determined for worms grazing on V. cholerae WT or Δ*mshA* mutant after the 2-h passage on E. coli OP50 (Fig. 3C). Despite a relatively high variation in colonization levels of WT and Δ*mshA* strains, worms grazing on V. cholerae WT showed significantly higher colonization than worms fed with the Δ*mshA* mutant. C. elegans grazing on V. cholerae WT showed a median colonization level of ~2 × 10^3^ CFU per worm, while nematodes grazing on the Δ*mshA* mutant exhibited a median colonization level of ~2 × 10^1^ CFU per worm. Taken together, the results indicate that MSHA is important for adherence of V. cholerae in the pharynx of C. elegans, which is an important step for successful colonization.

### MSHA does not contribute to the lethality of C. elegans.

As V. cholerae has been shown to cause lethality in C. elegans via secreted effectors, i.e., the hemolysin HlyA or the protease PrtV (12, 13), a potential contribution of MSHA to cause reduced longevity of C. elegans was investigated. Worms were raised on E. coli OP50 to reach the L4 stage before they were transferred onto plates harboring lawns of either E. coli OP50 or V. cholerae WT or *ΔmshA* mutant. In accordance with previous results (12, 13), we observed a marked decrease in the survival rate for the nematodes grazing on V. cholerae WT, with a median survival of approximately 5 days, compared to growth on E. coli OP50, with a median survival of approximately 14 days. As the worms exhibited similar survival dynamics while grazing on V. cholerae WT and on the *ΔmshA* mutant (Fig. S1), MSHA does not seem to be a dominant factor causing lethality in C. elegans.

10.1128/mSphereDirect.00238-18.1 FIG S1  MSHA does not contribute to the lethality of C. elegans grazing on V. cholerae. Shown is the survival in percent for L4-synchronized worms transferred on plates seeded with E. coli OP50 (dashed black line) or V. cholerae WT (dark gray solid line) or Δ*mshA* mutant (light gray solid line). Survival was monitored on days 1, 3, 5, 7, 9, and 11 starting with the transfer of L4 worms on day 0. Download FIG S1, TIF file, 1 MB. Copyright © 2018 List et al. 2018 List et al. This content is distributed under the terms of the Creative Commons Attribution 4.0 International license.

### MSHA contributes to developmental delay and growth retardation of C. elegans.

**FIG 5:**
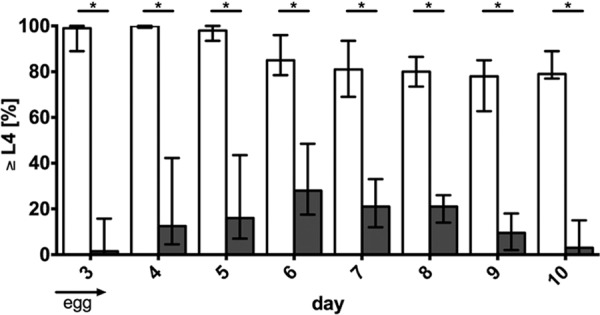
V. cholerae causes developmental delay in C. elegans. Development of C. elegans cultured on NGM agar plates containing E. coli OP50 (open bars) or V. cholerae WT (gray bars) was monitored for 10 days starting from egg stage on day 0. Shown are the median percentages of nematodes in L4 or later stages for day 3 to 10. The error bars indicate the interquartile ranges. Each data set consists of at least 10 independent experiments with an average of 30 worms per experiment. An asterisk above the respective data sets indicates a significant difference between the development of worms grazing on E. coli OP50 and those grazing on V. cholerae WT (*, *P* < 0.05 by Mann-Whitney U test).

Although survival was not altered between the V. cholerae strains, a disparity in the size of worms grazing on different bacterial strains throughout the routine microscopic evaluation of the survival assays was observed. This prompted us to analyze if MSHA of V. cholerae might be a crucial factor for developmental delay and reduced growth of C. elegans. Adult worms were allowed to lay eggs for a defined amount of time on plates harboring lawns of either E. coli OP50 or V. cholerae strains (see Materials and Methods for details). Subsequently, adult worms were removed and the development of C. elegans eggs to the L4 stage or beyond was monitored. In order to reveal the dynamics, development of C. elegans grazing on E. coli OP50 and V. cholerae WT was monitored for 10 days (Fig. 5). Almost all nematodes grazing on E. coli OP50 reached the L4 stage within 3 to 4 days. Within the following days, the percentage of worms in L4 or later stages declined modestly, which can be explained by the increasing death rate. In contrast to E. coli, the percentage of C. elegans in L4 or later stages was significantly lower for worms grazing on V. cholerae WT throughout the entire assay. In the case of V. cholerae, the number of worms in L4 or later stages peaked on day 6, followed by a steady decline with only a few worms surviving at day 10.

To exclude the possibility that E. coli OP50 provides better nutrition than V. cholerae, which might result in the observed developmental delay, the assay was repeated with heat-killed bacteria. Worms grazing on heat-killed V. cholerae WT or E. coli OP50 showed similar development, which was comparable to that of nematodes grazing on living E. coli OP50 (data not shown). Thus, V. cholerae seems to be an adequate nutrition source for C. elegans. In summary, development of nematodes cultured on living V. cholerae WT compared to those on E. coli OP50 was delayed and significantly reduced.

**FIG 6:**
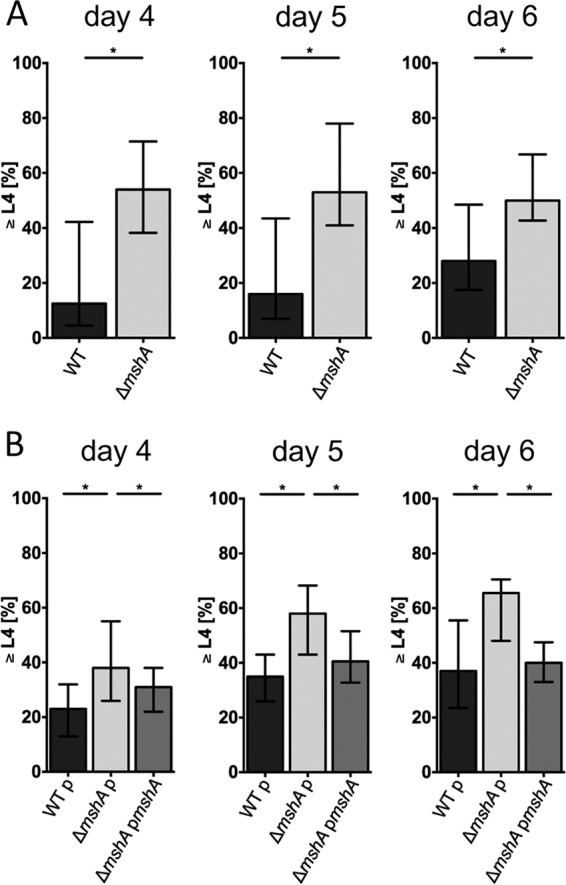
MSHA contributes to developmental delay of C. elegans grazing on V. cholerae. Development of C. elegans grazing on different V. cholerae strains (as indicated on the *x* axis) was monitored for 10 days starting from egg stage on day 0. Shown are the median percentages of nematodes in L4 or later stages for days 4, 5, and 6. The error bars indicate the interquartile ranges. Each data set consists of at least 8 independent experiments with an average of 30 worms per experiment. (A) Significant differences in the development of worms grazing on V. cholerae WT compared to those grazing on the Δ*mshA* mutant are indicated with an asterisk (*, *P* < 0.05 by Mann-Whitney U test). (B) Significant differences in the development of worms grazing on the Δ*mshA* mutant with empty vector compared to those grazing on V. cholerae WT with empty vector or complementation strain are indicated with an asterisk (*, *P* < 0.05 by Kruskal-Wallis test followed by *post hoc* Dunn’s multiple-comparison test).

Based on the developmental dynamics of nematodes grazing on V. cholerae WT, days 4, 5, and 6 were chosen for further comparative analyses of the WT and Δ*mshA* strains (Fig. 6A). For all time points, a significantly higher number of nematodes grazing on the Δ*mshA* strain reached the L4 stage compared to worms cultured on V. cholerae WT. While the presence of an empty vector in V. cholerae WT or the Δ*mshA* mutant had no effect on the observed differences in development of C. elegans, the enhanced development of worms grazing on the Δ*mshA* mutant was significantly decreased again upon worm cultivation on the Δ*mshA* mutant expressing *mshA* in *trans* on a complementation plasmid (Fig. 6B). Any effects on development by isopropyl-β-thiogalactopyranoside (IPTG) and ampicillin (Ap) can be excluded, as worms grazing on either regular NGM plates seeded with E. coli OP50 or NGM plates supplemented with IPTG/Ap and seeded with E. coli OP50 harboring an empty vector showed comparable developmental dynamics to the L4 stage (Fig. S2). Thus, the observed differences in C. elegans development can be attributed to the respective V. cholerae strain used.

10.1128/mSphereDirect.00238-18.2 FIG S2  IPTG and ampicillin (Ap) do not affect development of C. elegans. Development of C. elegans grazing on either regular NGM plates seeded with E. coli OP50 (dark gray) or NGM plates supplemented with IPTG/Ap and seeded with E. coli OP50 harboring the empty vector pMMB67EH (light gray) was monitored for 3 days starting from egg stage on day 0. Shown are the median percentages of nematodes in L4 or later stages for days 1, 2, and 3. The error bars indicate the interquartile range. Each data set consists of at least 8 independent experiments with an average of 30 worms per experiment. Download FIG S2, TIF file, 0.5 MB. Copyright © 2018 List et al. 2018 List et al. This content is distributed under the terms of the Creative Commons Attribution 4.0 International license.

**FIG 7:**
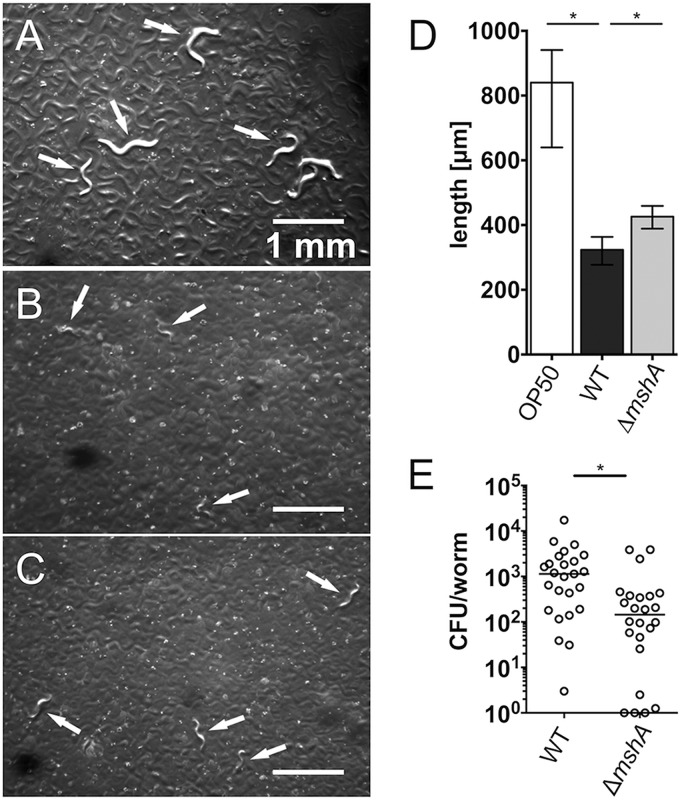
MSHA contributes to growth defect of C. elegans grazing on V. cholerae. (A to C) Images of plates with C. elegans grazing on E. coli OP50 (A) and V. cholerae WT (B) or Δ*mshA* (C) strain taken on day 4 of the developmental assay. Arrows mark representative worms on the plate. (D) Median length of C. elegans grazing on E. coli OP50 (open bar) or V. cholerae WT (dark gray bar) or Δ*mshA* mutant (light gray bar). The error bars indicate the interquartile ranges. Significant differences between the data sets are indicated with an asterisk (*, *P* < 0.05 by Kruskal-Wallis test followed by *post hoc* Dunn’s multiple-comparison test). (E) Colonization levels on day 4 of the developmental assay of worms which have been grazing on either V. cholerae WT or the Δ*mshA* mutant. Each circle represents the colonization level determined from 7 to 12 worms pooled for homogenization. The CFU were divided by the number of worms present in the respective homogenized pool to retrieve the CFU per worm (for details, see “Quantification of bacterial colonization” in Materials and Methods). The horizontal bar indicates the median of each data set. A significant difference is indicated by an asterisk (*, *P* < 0.05 by Mann-Whitney U test).

Furthermore, the length of the worms and their colonization with V. cholerae were comprehensively analyzed on day 4. Representative microscopic images of worms grazing on E. coli OP50 or the V. cholerae WT or Δ*mshA* strain are presented in Fig. 7A to C. C. elegans worms grazing on V. cholerae WT were significantly smaller than E. coli OP50-cultured worms (Fig. 7D). Consistent with the developmental assay, nematodes cultivated on the Δ*mshA* strain were significantly larger than those cultivated on V. cholerae WT, demonstrating that the absence of MSHA partially negates the developmental delay and growth retardation of C. elegans grazing on V. cholerae. In addition, a GFP-expressing variant of V. cholerae WT was used to analyze the spatial distribution in the worm at day 4 of the developmental assay (Fig. S3). A marked heterogeneity in the localization could be observed. The majority of worms showed detectable levels in the pharynx (Fig. S3), which is consistent with the localization in L4-synchronized worms cultivated for 1 day on V. cholerae WT pgfp (Fig. 4). However, several worms also exhibited fluorescence in the lower gut (Fig. S3F and I), suggesting that V. cholerae can spread to other parts of the gut at later time points. In some cases, the fluorescent signal seemed to be blurred. Thus, we cannot exclude the possibility that some fluorescence originates from GFP released from lysed V. cholerae. It should be noted that the analysis was hampered by the fragility of worms, which resulted in lysis and disintegration of the worms. An example of a damaged worm is given in Fig. S3J, K, and L. The prolonged cultivation on V. cholerae obviously causes the worms to become highly fragile for preparation for microscopy. Finally, bacterial colonization of C. elegans grazing for 4 days on the V. cholerae WT or Δ*mshA* strain was quantified by CFU plating. Worms grazing on V. cholerae WT also showed significantly higher colonization than worms fed with the Δ*mshA* mutant (Fig. 7E), which is consistent with the colonization data for L4-synchronized worms (Fig. 3C). The colonization level observed on day 4 of the developmental assays was slightly lower than that of L4-stage worms, which might be due to the delayed development and growth retardation of worms fed on V. cholerae.

10.1128/mSphereDirect.00238-18.3 FIG S3  Localization of V. cholerae in C. elegans at day 4 during developmental assay. Four representative epifluorescence microscopy images of C. elegans grazing on plates with V. cholerae WT pgfp for 4 days starting from egg stage on day 0. Worms were passaged via E. coli OP50 for 2 h before microscopy to remove loosely attached V. cholerae. GFP signal was detected in the green channel (excitation of 400 nm, emission of 500 to 550 nm). Shown are bright-field images (A, D, G, and J), fluorescent images (B, E, H, and K), and the merged images (C, F, I, and L). Download FIG S3, TIF file, 2.8 MB. Copyright © 2018 List et al. 2018 List et al. This content is distributed under the terms of the Creative Commons Attribution 4.0 International license.

## DISCUSSION

It has become evident that clinically relevant V. cholerae persists in the aquatic reservoir between devastating outbreaks (9, 10). It is essential that bacterial load is maintained at a certain level sufficient to cause a new outbreak. This requires several survival strategies of V. cholerae to cope with the harsh conditions of an aquatic lifestyle, facing, for example, temperature shifts, changes in osmolarity, and nutrient limitation. Biofilm formation, chitin utilization, and the entrance into a VBNC status are known mechanisms of V. cholerae to enhance persistence in the aquatic reservoir. Especially during colder periods, the sediments of the aquatic ecosystem act as a reservoir for V. cholerae (24, 25). Interestingly, bacterivorous nematodes are highly abundant in the sediment, which emphasizes that V. cholerae is under high grazing pressure and top-down control by these predators (10, 24, 26–28). Recent studies introduced the nematode C. elegans, which has become an important model organism for genetic and developmental studies, as a natural predator model for V. cholerae (12, 13). This nematode is an ideal choice as it can be easily maintained under laboratory conditions with a variety of mutants and assays available. Both abovementioned studies demonstrated that V. cholerae can reduce the life span of C. elegans and identified two secreted effectors, the protease PrtV and the hemolysin HlyA, which contribute to the observed lethality (12, 13). These reports imply that the facultative human pathogen has evolved responses and defense mechanisms to counteract bacterivorous nematodes.

In order to gain insights into these bacterial strategies, we focused on the identification of V. cholerae genes upregulated upon exposure to C. elegans in the present study. Therefore, we used the well-established resolvase-based reporter gene system RIVET, which allows detection of spatiotemporal gene expression even in diverse populations (16, 29–31). In contrast to alternative techniques like microarrays, transcriptome sequencing (RNA-seq), or reverse transcription-quantitative PCR (qRT-PCR), this single-cell-based expression technology avoids purification of bacterial RNA in decent quality and quantity, requires relatively small amounts of bacteria, and even allows the identification of genes that are only transiently induced, as the excision of the reporter gene cassette is irreversible. Thus, the resolvase-based reporter gene system is ideally suited for a first study identifying V. cholerae genes in C. elegans. It should be noted that the presented screen does not claim to be saturated. Consequently, several other upregulated genes might have been missed and still remain to be identified.

In total, we were able to identify 109 *aec* genes. An advantage of the resolvase-based reporter gene system is the ability to recover the integrated suicide plasmids harboring the induced *tnpR* fusion and to subsequently reconstruct the original unresolved strain. These reconstructed strains can be tested for their resolution frequency under control conditions as well as during colonization of C. elegans. A validation revealed that 7 out of 10 reconstructed *tnpR* fusion strains showed a significantly higher resolution frequency during colonization of C. elegans than under the control condition. This corresponds to an acceptable accuracy rate of 70%.

Gene ontology analysis revealed that the genes involved in metabolic processes and biological regulation are enriched within the *aec* genes, indicating that V. cholerae alters its metabolism and regulatory pathways upon exposure to the worm. Among the identified *aec* genes were 5 relevant for adhesion to and degradation of chitin. Two of these, VCA0027, encoding a putative chitinase, and VC0414, encoding a biogenesis protein of the MSHA type IV pilus, were included in the validation and demonstrated a significant induction upon exposure to C. elegans. Within this study, we selected the MSHA type IV pilus for further characterization and analyzed its impact on longevity, growth, and development of C. elegans, which are affected by V. cholerae. Based on the data presented here, MSHA has no effect on physiology of the worm. Instead, MSHA has previously been shown to be important for the first steps in biofilm formation by facilitating adherence to abiotic surfaces, like borosilicate, as well as biotic surfaces, like zooplankton and crab shells (19, 32, 33). In concordance with these reports, we can demonstrate that MSHA promotes attachment and colonization of the C. elegans pharynx, which contains chitin on interior regions (34). Based on the current data, it is unclear whether MSHA specifically interacts with chitin or enhances chitin adherence in a surface chemistry-independent manner. Meibom et al. suggested that MSHA could also facilitate adherence to the epicuticle, which naturally covers biotic chitinous surfaces and therefore represents the outermost layer (35). Such an epicuticle would also be present in the pharynx of C. elegans, thought to be secreted by pharyngeal gland cells (http://www.wormbook.org/chapters/www_cuticle/cuticle.html). In fact, a deletion mutant of *gbpA*, encoding an alternative chitin-binding protein with specificity for *N*-acetylglucosamine oligosaccharides (36), was also analyzed in this study but showed no difference for C. elegans growth or development compared to the V. cholerae WT (see Fig. S4 in the supplemental material). Together, these results could indicate that initial attachment of V. cholerae in the pharynx is chitin independent but that the interaction could be reinforced subsequently in a chitin-dependent manner.

10.1128/mSphereDirect.00238-18.4 FIG S4  GbpA does not contribute to developmental delay of C. elegans grazing on V. cholerae. Development of C. elegans grazing on V. cholerae WT or Δ*gbpA* mutant was monitored starting from egg stage on day 0. Shown are the median percentages of nematodes in L4 or later stages for days 4, 5, and 6. The error bars indicate the interquartile range. Each data set consists of at least 8 independent experiments with an average of 30 worms per experiment. Download FIG S4, TIF file, 2.7 MB. Copyright © 2018 List et al. 2018 List et al. This content is distributed under the terms of the Creative Commons Attribution 4.0 International license.

How can MSHA impact growth and development of C. elegans without affecting longevity? Lethality for C. elegans by V. cholerae can be attributed to at least two secreted effectors, PrtV and HlyA. One could speculate that they act very potently and/or fast on C. elegans. As both effectors are secreted, they could also be constantly ingested by C. elegans from the medium in addition to the amounts secreted by V. cholerae colonizing the worm. Thus, a stable colonization by V. cholerae resulting in a long exposure to the effectors is not required to reduce the longevity of C. elegans. Alternatively, V. cholerae expresses PrtV and HlyA independently of C. elegans and can affect the worm directly upon first contact. Notably, neither *prtV* nor *hlyA* was identified as an *aec* gene. While lethality is a toxigenic effect, delays in development might be due to poor nutrition. Bacterial colonization in the pharynx might block ingestion of food. Alternatively, colonizing V. cholerae could deplete specific nutrients which are essential for C. elegans development. Furthermore, at least one annotated chitinase (VCA0027) has been identified and validated as a bona fide *aec* gene. Thus, it can be hypothesized that the chitin utilization program of V. cholerae is induced upon exposure to C. elegans. This will slowly but steadily result in chitin degradation on the pharyngeal luminal surfaces and thereby affect the growth and development of the worm. Future studies are currently directed to dissecting the role of the five predicted chitinases encoded by V. cholerae.

Altogether, this study provides a first glimpse into the response of V. cholerae upon exposure to C. elegans. In addition, MSHA was characterized as a crucial factor for attachment and colonization of the C. elegans pharynx, which results in growth retardation and developmental delay of C. elegans.

## MATERIALS AND METHODS

### Bacterial strains, plasmids, and growth conditions.

Bacterial strains and plasmids used in this study are listed in Table S2 in the supplemental material; oligonucleotides are listed in Table S3. V. cholerae AC53, a spontaneous streptomycin (Sm)-resistant mutant of the clinical isolate E7946 (O1 El Tor Ogawa), was used as the wild-type (WT) strain. *E. coli* strain OP50 served as a general food source for C. elegans, while E. coli strains DH5αλ*pir* and SM10λ*pir* were used for genetic manipulations. If not noted otherwise, bacterial strains were cultured in Luria-Bertani (LB) broth or on LB broth agar plates with aeration at 37°C. Antibiotics and other supplements were used in the final concentrations indicated: streptomycin (Sm), 100 µg/ml; kanamycin (Kn), 50 µg/ml; Ap, 100 µg/ml or 50 µg/ml in combination with a second antibiotic and in all assays using NGM agar plates; chloramphenicol (Cm), 30 µg/ml (E. coli) or 2 µg/ml (V. cholerae); IPTG, 0.5 mM; glucose (Glc), 0.2%; sucrose (Suc), 10%; 5-bromo-4-chloro-3-indolyl-β-d-galactopyranoside (X-Gal), 30 µg/ml.

10.1128/mSphereDirect.00238-18.6 TABLE S2  Bacterial strains and plasmids used in the study. Download TABLE S2, DOCX file, 0.03 MB. Copyright © 2018 List et al. 2018 List et al. This content is distributed under the terms of the Creative Commons Attribution 4.0 International license.

10.1128/mSphereDirect.00238-18.7 TABLE S3  Oligonucleotides used in this study. Download TABLE S3, DOCX file, 0.02 MB. Copyright © 2018 List et al. 2018 List et al. This content is distributed under the terms of the Creative Commons Attribution 4.0 International license.

### Caenorhabditis elegans maintenance.

C. elegans strains Bristol N2 and SS104 (see Table S2) were obtained from the Caenorhabditis Genetics Center and routinely maintained at 24°C (Bristol N2) or 16°C (SS104) on nematode growth medium (NGM) agar plates with E. coli strain OP50 by standard methods (37, 38). C. elegans strain Bristol N2 was used for the RICET screen, while C. elegans strain SS104, a temperature-sensitive mutant that produces progeny at temperatures of ≤16°C but not at temperatures of ≥24°C, was used for all other experiments to allow synchronization of the worms. Synchronization was achieved by transferring C. elegans SS104 cultivated at 16°C onto NGM agar plates for 2 h. After 2 h of egg laying, adult worms were removed, and incubation of the plates at 24°C allowed the hatched progeny to reach the L4 stage within 3 days.

### Genetic manipulations and construction of plasmids.

The isolation of chromosomal DNA, PCRs, the purification of plasmids or PCR products, the construction of suicide and expression plasmids, and the subsequent generation of deletion mutants were carried out as described previously (39). QIAquick gel extraction and QIAquick PCR purification kits (Qiagen) were used for purifying PCR products and digesting plasmid DNA. PCRs for subcloning were carried out using the Q5 high-fidelity DNA polymerase (New England BioLabs [NEB]), while *Taq* DNA polymerase (NEB) was used for all other PCRs. The Δ*mshA* and Δ*gbpA* in-frame deletion mutants were constructed according to the method of Donnenberg and Kaper (40) using the suicide vector pCVD442. The respective suicide vectors pCVD442ΔmshA and pCVD442ΔgbpA were constructed by PCR amplification of approximately 800-bp fragments, representing upstream and downstream regions of the gene of interest, using the oligonucleotide pairs y-x_1 and y-x_2 or y-x_3 and y-x_4, where y represents the gene (*mshA* or *gbpA*) and x represents the restriction site/enzyme used (Table S3). These upstream/downstream fragments were digested with the appropriate restriction enzyme indicated by the name of the oligonucleotide and ligated into an identically digested suicide plasmid, pCVD442. The respective suicide plasmids were first transformed into E. coli Sm10λ*pir* and then transferred into V. cholerae via conjugation. Conjugants were purified on Sm- and Ap-containing agar plates to select for the integration of the plasmid into the V. cholerae chromosome, followed by growth on sucrose to select for the second recombination and obtain Ap^s^ colonies, in which an excision of the plasmid from the chromosome took place. The correct deletions were confirmed by PCR (data not shown). For construction of the expression plasmid pmshA, the oligonucleotides VC0409-SacI_fw and VC0409-XbaI_rv were used to amplify the respective gene (Table S3). The resulting PCR fragment was digested with the appropriate restriction enzymes and ligated into an identically digested IPTG-inducible plasmid, pMMB67EH. Ligation products were transformed into DH5αλ*pir*, and Ap^r^ colonies were characterized by PCR for the correct construct (data not shown).

### Screening for *aec* genes.

**FIG 8:**
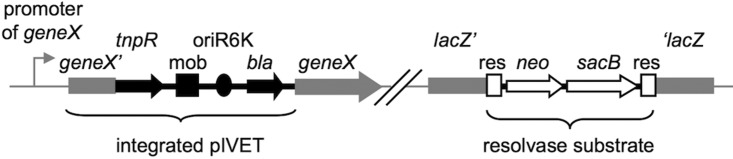
Illustration of the genetic components of RIVET. Chromosomal sequences are in gray, pIVET parts are in black, and the res cassette parts are in open shapes. The suicide vector pIVET is integrated into V. cholerae hypothetical *geneX* via homologous recombination, resulting in a merodiploid state in which *geneX* and *tnpR* (resolvase) are transcriptionally fused and controlled by the chromosomal promoter of *geneX*. The mobilization (mob), origin of replication (oriR6K), and Ap^r^ (*bla*) regions of pIVET, as well as the genes for Kn^r^ (*neo*) and Suc^s^ (*sacB*) and the target sites of resolvase (res) of the res cassette, are indicated.

A slightly adapted version of the recombination-based *in vivo* expression technology (RIVET, Fig. 8) in combination with a library of 12,000 random transcriptional gene-*tnpR* fusions (16, 41) was used to identify genes induced during colonization of C. elegans, which was consequently renamed recombination-based in C. elegans expression technology (RICET). The original gene-*tnpR* fusion library was generated to identify genes induced during colonization of the mouse model and consequently constructed using LB broth at 37°C. To minimize the identification of differentially expressed genes solely caused by the specific growth requirements of C. elegans, the entire gene-*tnpR* fusion library was passaged once on NGM plates at 22°C prior to the screening for *aec* genes. Briefly, each pool was spread on NGM plates and incubated overnight at 22°C before the bacteria were collected from the NGM plate and purified on LB-Ap/Sm/Kn plates to remove all resolved strains. Finally, the NGM-passaged library was refrozen in 12 purified pools containing ~5,000 colonies (Ap^r^/Sm^r^/Kn^r^) each. For the screening of *aec* genes, an aliquot of each pool of the library was spread in triplicate on LB-Ap/Sm/Kn plates, resuspended in LB broth, and adjusted to an optical density at 600 nm (OD_600_) of 0.1. One hundred microliters of the suspension was spread in the middle of an NGM agar plate in a rectangular shape using a Drigalski spatula. After incubation overnight at 22°C, ~60 synchronized young adult worms were transferred onto the plate and allowed to graze for 16 h at 22°C. To remove bacteria attached on the outside of the nematode, the worms were then transferred onto an empty NGM agar plate for 1 h followed by a 1-min dip in 30% ethanol. Finally, batches of ~20 worms were collected in 1 ml saline, briefly vortexed, and homogenized using glass beads (1 mm in diameter; Roth) in combination with a PowerLyzer 24 (Mo Bio Laboratories, Inc.), applying 2,000 rpm for 1 min. Efficient removal of bacteria attached on the outside of the nematode and lysis of C. elegans worms were monitored by plating appropriate dilutions directly before and after homogenization on LB-Sm agar plates. Throughout the screen, less than 10 CFU/worm was observed before homogenization, while after homogenization ~10^3^ CFU/worm could be detected (data not shown). Serial dilutions were plated on Sm/Suc agar plates (1% tryptone, 0.5% yeast extract, 1.6% agar) to select for resolved strains. After a 2-day incubation at room temperature (RT), colonies were picked, patched in parallel on LB-Sm/Ap and LB-Sm/Km agar plates, and incubated overnight at 37°C. Ap^r^ and Km^s^ clones were screened for diverse gene-*tnpR* fusions via different PCR product sizes using the oligonucleotides IVET-1 and IVET-2 as described previously (37). The plasmids containing the gene-*tnpR* fusion (pIVETs) of 5 to 8 different resolved strains were recovered (Qiagen Spin Miniprep kit), and aliquots were sent to Agowa Genomics for sequencing with the oligonucleotide IVET-3 as previously described (17, 42). Sequences were compared to the V. cholerae N16961 genome database at the J. Craig Venter Institute with BLASTn (http://blast.jcvi.org/cmr-blast/). Gene annotations and operon predictions shown in Table S1 were determined by the KEGG database (http://www.genome.jp/kegg/) and the operon prediction database (http://biocomputo2.ibt.unam.mx/OperonPredictor/), respectively (43–46). Transcriptional fusions to any annotated open reading frame (ORF) within which *tnpR* had inserted in the same orientation were considered as described previously (16).

### Reconstruction of fusion strains and determination of the resolution frequency.

The reconstruction of unresolved V. cholerae fusion strains was carried out as described previously (38). For all resolution frequency quantifications, strains were grown overnight on LB-Sm/Ap/Km agar plates, resuspended in LB broth, and adjusted to an OD_600_ of 5. Fifty microliters of this inoculum was spread in the middle of an NGM plate in a rectangular shape using a Drigalski spatula and air dried for a few minutes. To determine the resolution frequency *in vitro* (control condition), plates were incubated for 16 h at 22°C before bacteria were harvested and resuspended in LB broth. To determine the resolution frequency in C. elegans, ~60 synchronized young adult worms were transferred onto the plate and allowed to graze for 16 h at 22°C before the worms were purified from bacteria attached to the outside and homogenized as described above (see “Screening for *aec* genes”). The resolution frequencies *in vitro* as well as in C. elegans were determined by plating serial dilutions on LB-Sm/Ap and LB-Sm/Km agar plates. The resolution frequency is expressed in percent and calculated as follows: (Sm/Ap CFU − Sm/Km CFU)/(Sm/Ap CFU × 100), as described previously (16).

### C. elegans survival assay.

Survival assays were essentially performed as previously described (12, 13). Respective V. cholerae strains or E. coli OP50 was incubated at 37°C overnight in LB broth and adjusted to an OD_600_ of 1, and 100 µl was spread on an NGM agar plate in a rectangular shape using a Drigalski spatula. After incubation overnight at 24°C, ~30 synchronized L4-stage C. elegans SS104 worms were transferred onto the plates. Plate were subsequently incubated at 24°C and scored for live worms every day. A worm was considered dead when it no longer responded to touch. Any worms that died as a result of migrating outside the bacterial rectangular lawn and getting stuck to the wall of the plate were excluded from the analysis.

### C. elegans developmental assay.

Respective V. cholerae strains or E. coli OP50 was incubated at 37°C overnight in LB broth and adjusted to an OD_600_ of 1, and 100 µl was spread on an NGM agar plate in a rectangular shape using a Drigalski spatula. In the case of strains harboring pMMB67EH or derivatives, NGM plates supplemented with IPTG (0.5 mM) and Ap (100 µg/ml) were used. After incubation overnight at 24°C, ~20 synchronized young adult C. elegans SS104 worms were transferred onto the plate and allowed to lay eggs for 2 h before they were removed. This was defined as day 0, was allowed to control synchronized hatching, and resulted in similar numbers of eggs on different NGM agar plates (~30 eggs/plate). Plates were incubated at 24°C, and development into at least the L4 stage was monitored every 24 h for 10 days. The L4 stage was chosen as the criterion as it can be easily distinguished via microscopy from earlier stages by the presence of a vulva. Data are presented as percentage of worms in the L4 stage or later stages divided by the total number of live worms present on day 3. All worms moving or responding to touch were considered to be alive. During the developmental assay, the length of the worms was determined on day 4 using the ImageJ software from images of living worms, and colonization levels were quantified as described below (see “Quantification of bacterial colonization” for details).

### Microscopy.

For routine handling, developmental stage evaluation, and length determination of C. elegans, the Leica M60 or Motic SMZ-140-N2GG stereomicroscope was used. Nematodes on NGM plates were imaged using the Leica M60 stereomicroscope in combination with a Tucsen 3.0 MP microscope USB camera. Localization of V. cholerae in C. elegans was investigated by fluorescence microscopy using a Nikon Eclipse Ti-E inverted microscope equipped with a Nikon DS-Qi2 camera and Nikon Plan Fluor 40× objective (numerical aperture [NA], 1.30). All images were analyzed by Nis-Elements BR version 4.30.02 software. For localization studies, synchronized L4-stage C. elegans SS104 worms were passaged via NGM agar plates without bacteria to fast for approximately 4 h, before they were transferred onto NGM agar plates containing a rectangular lawn of GFP^+^ bacteria (prepared as described above) and incubated for 20 h at 24°C. Afterward, the nematodes were either directly prepared for microscopy or transferred to NGM agar plates seeded with E. coli OP50 without GFP expression for another 2 h to remove loosely attached bacteria inside the worms prior to microscopy. Alternatively, worms were allowed to lay eggs on NGM agar plates seeded with V. cholerae pgfp, and plates were subsequently incubated at 24°C for 4 days. Prior to microscopy, worms were transferred to NGM agar plates seeded with E. coli OP50 for 2 h. Subsequently, worms were immobilized using NaN_3_ (0.01%) and embedded in fluorescent mounting agent (Dako). Presence and localization of V. cholerae in C. elegans were investigated by fluorescence microscopy using a Nikon Eclipse Ti-E inverted microscope equipped with a Nikon DS-Qi2 camera and Nikon Plan Fluor 10× (NA, 0.3) or 40× (NA, 1.30) objective. GFP was detected using an excitation of 400 nm and emission of 500 to 550 nm. All images were analyzed by Nis-Elements BR version 4.30.02 software. A signal intensity inside the worm at least 10-fold above background (surrounding the worm) was defined as positive signal. The statistical significance of associations between the incidences of positive signals observed in C. elegans grazing on either V. cholerae WT or mutant was assessed by the odds ratio using the Fisher exact test (Prism5; GraphPad Software, USA).

### Quantification of bacterial colonization.

Colonization was determined for living C. elegans SS104 worms on day 4 of the developmental assay (see “C. elegans developmental assay”) or for synchronized L4-stage C. elegans SS104 worms grazing for 20 h on V. cholerae WT or Δ*mshA* mutant (see “Microscopy”). Hence, worms were allowed to lay eggs on NGM agar plates seeded with either V. cholerae WT or Δ*mshA* mutant, and plates were subsequently incubated at 24°C for 4 days. Alternatively, worms were synchronized on E. coli OP50 for 3 days and subsequently transferred onto NGM agar plates seeded with V. cholerae WT or Δ*mshA* mutant for 20 h.

In all cases, worms grazing on V. cholerae were transferred to NGM agar plates seeded with E. coli OP50 for another 2 h and subsequently onto an empty NGM plate for 2 min to remove loosely attached bacteria, followed by a 1-min dip in 30% ethanol to eliminate bacteria attached to the exterior surface of the worm. Then, 7 to 12 worms were collected in 1 ml LB broth, briefly vortexed, and homogenized using glass beads (1 mm in diameter; Roth) in combination with a PowerLyzer benchtop bead-based homogenizer (Mo Bio Laboratories, Inc.), applying 2,000 rpm for 1 min. Remaining bacteria attached on the outside of the nematode were monitored by plating appropriate dilutions directly before homogenization on LB/Sm agar plates, while colonization levels of the worms were determined by plating appropriate dilutions of the homogenized worms on LB/Sm agar plates. In all cases, the CFU before homogenization was at least 10-fold lower than the CFU after homogenization, suggesting efficient removal of bacteria attached to the exterior of the worm. Results are given by CFU/worm calculated as follows: (CFU after homogenization − CFU before homogenization)/number of worms.

### Statistical analysis.

Unless stated otherwise, the data are presented as medians with interquartile ranges. Data were analyzed using the Mann-Whitney U test for single comparisons or the Kruskal-Wallis test followed by *post hoc* Dunn’s multiple-comparison test, and differences were considered significant for *P* values of <0.05.
