# Supplementary material for: Genes Activated by Vibrio cholerae upon Exposure to Caenorhabditis elegans Reveal the Mannose-Sensitive Hemagglutinin To Be Essential for Colonization
Source: mSphere. 2018 May 23;3(3):e00238-18. doi: 10.1128/mSphereDirect.00238-18 (PMC5967197; doi:10.1128/mSphereDirect.00238-18)
Supplement: TABLE S1 [file sph003182553st1.docx]

**TABLE S1: *V. cholerae* genes activated upon exposure to *C. elegans***

| **Gene Locus^a^** | **Operon^b^** | **Annotation/Gene Symbol^a^** |
| --- | --- | --- |
| VC0024 |  | sulfur transfer protein SirA |
| VC0026 |  | zinc-binding alcohol dehydrogenase |
| VC0051 | VC0051-2 | phosphoribosylaminoimidazole carboxylase ATPase subunit (EC:4.1.1.21) |
| VC0065 | VC0061-6 | thiazole synthase ThiG |
| VC0066 | VC0061-6 | thiamine biosynthesis protein ThiH |
| VC0071 |  | DNA-binding transcriptional regulator AsnC |
| VC0072 | VC0072-3 | sensory box/GGDEF family protein |
| VC0080 |  | hypothetical protein |
| VC0081 |  | hypothetical protein |
| VC0089 |  | cytochrome c551 peroxidase |
| VC0117 | VC0117-20 | protoheme IX synthesis protein HemY |
| VC0121 |  | hypothetical protein |
| VC0128 | VC0124-9 | site-specific tyrosine recombinase XerC |
| VC0129 | VC0124-9 | hypothetical protein |
| VC0130 |  | GGDEF family protein |
| VC0132 | VC0131-2 | hypothetical protein |
| VC0135 |  | lysophospholipase L2 |
| VC0136 | VC0136-7 | *rhtB*; homoserine/homoserine lactone efflux protein |
| VC0184 | VC0183-6 | hypothetical protein |
| VC0194 | VC0191-4 | gamma-glutamyltranspeptidase |
| VC0207 | VC0205-7 | *murP*; PTS system N-acetylmuramic acid transporter subunits IIBC |
| VC0283 |  | hypothetical protein |
| VC0299 | VC0299-301 | DNA polymerase III subunit epsilon |
| VC0300 | VC0299-301 | hypothetical protein |
| VC0303 | VC0302-3 | sensor histidine kinase |
| VC0338 |  | transporter |
| VC0376 |  | hypothetical protein |
| VC0386 | VC0384-6 | phosphoadenosine phosphosulfate reductase (EC:1.8.4.8) |
| VC0392 |  | aminotransferase, class V |
| VC0401 | VC0399-413 | MSHA biogenesis protein MshL |
| VC0412 | VC0399-413 | MSHA biogenesis protein MshO |
| VC0414 | VC0414 | MSHA biogenesis protein MshQ |
| VC0440 |  | dihydrofolate reductase (*folA*) (operon mit VC0438 (cons. Hyp.)- VC0440 ) |
| VC0606 |  | nitrogen regulatory protein P-II |
| VC0622 |  | sensory box sensor histidine kinase/response regulator ChiS |
| VC0667 |  | aldo/keto reductase family oxidoreductase TAS |
| VC0974 | VC0973-4 | MerR family transcriptional regulator |
| VC0998 |  | hypothetical protein |
| VC1008 |  | sodium-type flagellar protein MotY |
| VC1134 | VC1132-9 | histidinol-phosphate aminotransferase (EC:2.6.1.9) |
| VC1169 | VC1169-76 | trpA; tryptophan synthase subunit alpha (EC:4.2.1.20) |
| VC1268 | VC1268-70 | hypothetical protein |
| VC1317 | VC1317-8 | hypothetical protein |
| VC1338 | VC1335-9 | *acnA*; aconitate hydratase (EC:4.2.1.3) |
| VC1351 |  | hypothetical protein |
| VC1389 |  | hypothetical protein |
| VC1495 |  | hypothetical protein |
| VC1644 |  | hypothetical protein |
| VC1817 |  | sigma-54 dependent transcriptional regulator |
| VC1827 | VC1826-7 | *manA-2*; mannose-6-phosphate isomerase |
| VC1861 | VC1861-4 | amino acid ABC transporter, permease protein |
| VC1927 | VC1927-9 | *dctD-1*; C4-dicarboxylate transport protein |
| VC2067 | VC2058-69 | MinD-related protein |
| VC2133 | VC2120-37 | flagellar MS-ring protein FliF |
| VC2209 |  | nonribosomal peptide synthetase VibF |
| VC2247 | VC2244-60 | *lpxB*; lipid-A-disaccharide synthase (EC:2.4.1.182) |
| VC2264 |  | hypothetical protein |
| VC2319 | VC2319-20 | exodeoxyribonuclease V, 67 kDa subunit |
| VC2338 |  | *lacZ*, authentic frameshift |
| VC2376 | VC2376-7 | gltB; glutamate synthase subunit alpha (EC:1.4.1.13) |
| VC2438 |  | bifunctional glutamine-synthetaseadenylyltransferase/deadenyltransferase (EC:2.7.7.42) |
| VC2452 | VC2451-2 | 23S rRNA 5-methyluridine methyltransferase |
| VC2454 |  | GGDEF family protein |
| VC2464 | VC2464-7 | sigma-E factor regulatory protein RseC |
| VC2474 | VC2474-7 | 2-octaprenyl-6-methoxyphenol hydroxylase |
| VC2481 | VC2480-1 | D-3-phosphoglycerate dehydrogenase |
| VC2504 | VC2504-6 | D-lactate dehydrogenase |
| VC2560 | VC2558-61 | sulfate adenylyltransferase subunit 2 (EC:2.7.7.4) |
| VC2605 | VC2605-7 | hypothetical protein |
| VC2621 |  | extracellular nuclease-related protein |
| VC2641 | VC2641-4 | *argH*; argininosuccinate lyase (EC:4.3.2.1) |
| VC2649 |  | *cysE*; serine acetyltransferase (EC:2.3.1.30) |
| VC2652 |  | hypothetical protein |
| VC2671 |  | hypothetical protein |
| VC2700 |  | pullulanase *pulA*, authentic frameshift |
| VC2705 |  | sodium/solute symporter, putative |
| VC2713 | VC2713-4 | osmolarity sensor protein EnvZ |
| VC2717 |  | hypothetical protein |
| VC2725 | VC2723-32 | general secretion pathway protein L |
| VC2739 |  | hypothetical protein |
| VC2750 | VC2748-50 | GGDEF family protein |
| VC2757 | VC2752-7 | hypothetical protein |
| VC2759 | VC2758-9 | 3-ketoacyl-CoA thiolase (EC:2.3.1.16) |
| VCA0002 |  | hypothetical protein |
| VCA0027 |  | chitinase (chiA-2) |
| VCA0046 |  | hypothetical protein |
| VCA0065 | VCA0063-7 | hypothetical protein |
| VCA0117 | VCA0107-17 | sigma-54 dependent transcriptional regulator |
| VCA0142 |  | C4-dicarboxylate transport transcriptional regulator |
| VCA0211 |  | sensory box sensor histidine kinase |
| VCA0544 |  | hypothetical protein |
| VCA0684 | VCA0682-4 | regulatory protein UhpC |
| VCA0686 | VCA0685-7 | iron(III) ABC transporter, permease protein |
| VCA0758 | VCA0757-60 | arginine transporter permease subunit ArtQ |
| VCA0766 | VCA0764-6 | cytochrome c554 |
| VCA0777 | VCA0776-8 | hypothetical protein |
| VCA0779 |  | hypothetical protein |
| VCA0849 |  | hypothetical protein |
| VCA0860 | VCA0860-1 | periplasmic alpha-amylase precursor |
| VCA0959 | VCA0959-60 | hypothetical protein |
| VCA0985 | VCA0984-5 | oxidoreductase/iron-sulfur cluster-binding protein |
| VCA0992 | VCA0992-3 | glutaredoxin |
| VCA0995 |  | hypothetical protein |
| VCA1015 |  | Na+/H+ antiporter |
| VCA1032 |  | hypothetical protein |
| VCA1053 |  | hypothetical protein |
| VCA1074 |  | AraC/XylS family transcriptional regulator |
| VCA1085 |  | hypothetical protein |
| VCA1092 | VCA1090-7 | methyl-accepting chemotaxis protein |

^a^Gene locus and Annotation/Gene symbol are according to Kyoto Encyclopedia of Genes and Genomes (http://www.genome.jp/kegg/) ([43-45](#_ENREF_43))

^b^Operon prediction according to http://operons.ibt.unam.mx/OperonPredictor/ (Taboada B, Ciria R, Martinez-Guerrero CE, Merino E. 2012. ProOpDB: Prokaryotic Operon DataBase. Nucleic Acids Research 40:D627-D631.)
