# Supplementary figures and images for: Genes Activated by Vibrio cholerae upon Exposure to Caenorhabditis elegans Reveal the Mannose-Sensitive Hemagglutinin To Be Essential for Colonization
Source: mSphere. 2018 May 23;3(3):e00238-18. doi: 10.1128/mSphereDirect.00238-18 (PMC5967197; doi:10.1128/mSphereDirect.00238-18)

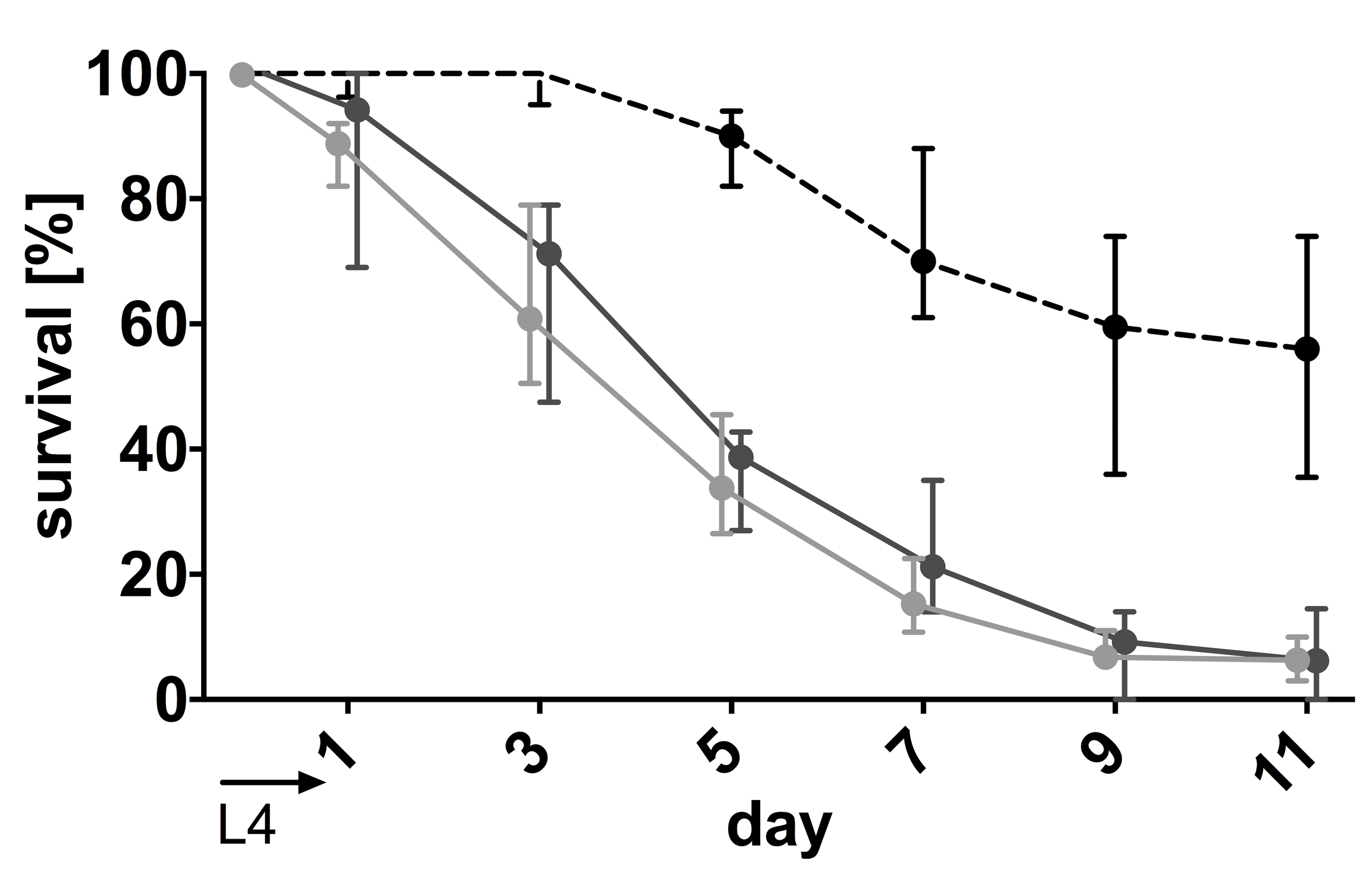

Supplement: FIG S1 [file sph003182553sf1.tif]

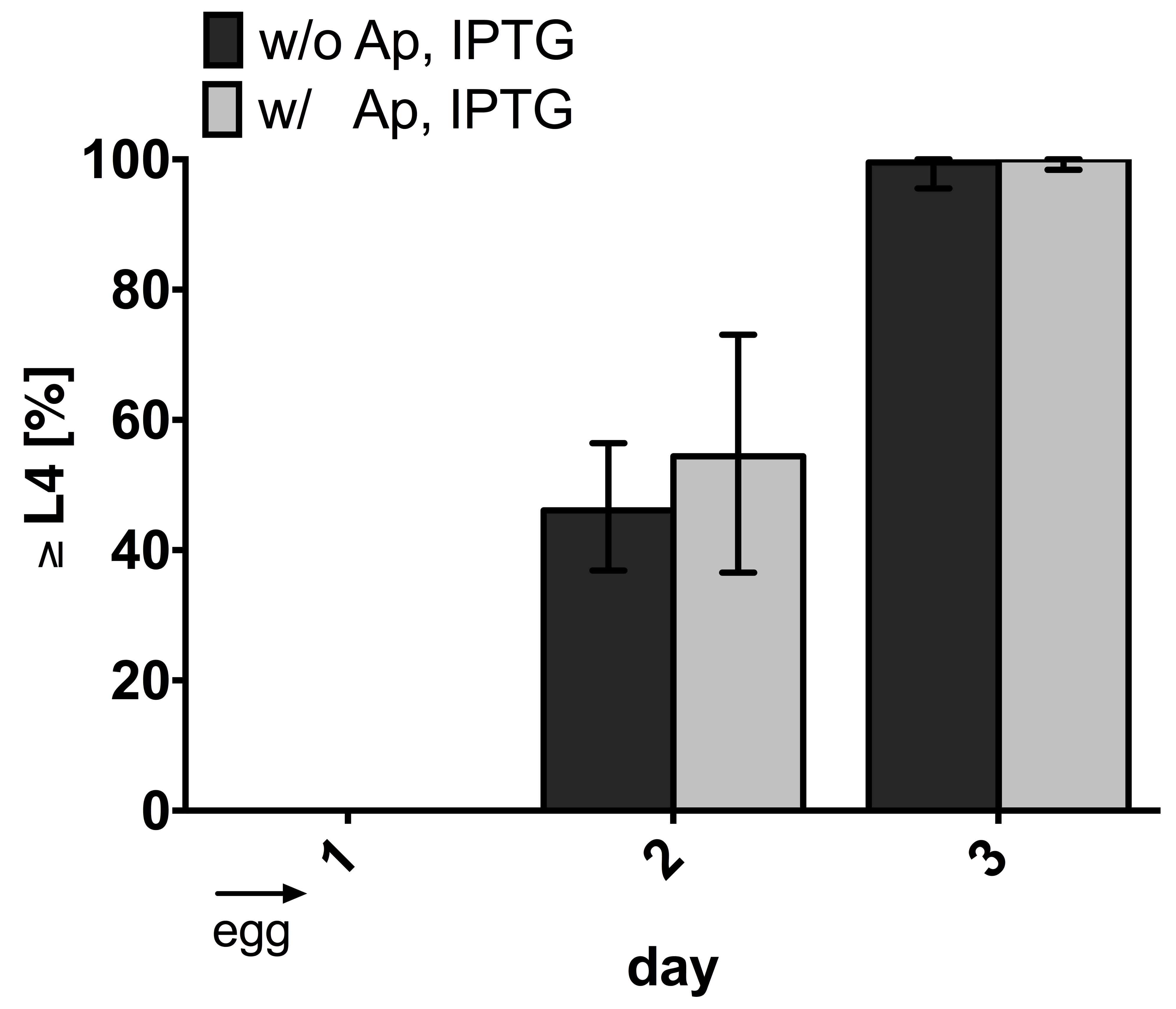

Supplement: FIG S2 [file sph003182553sf2.tif]

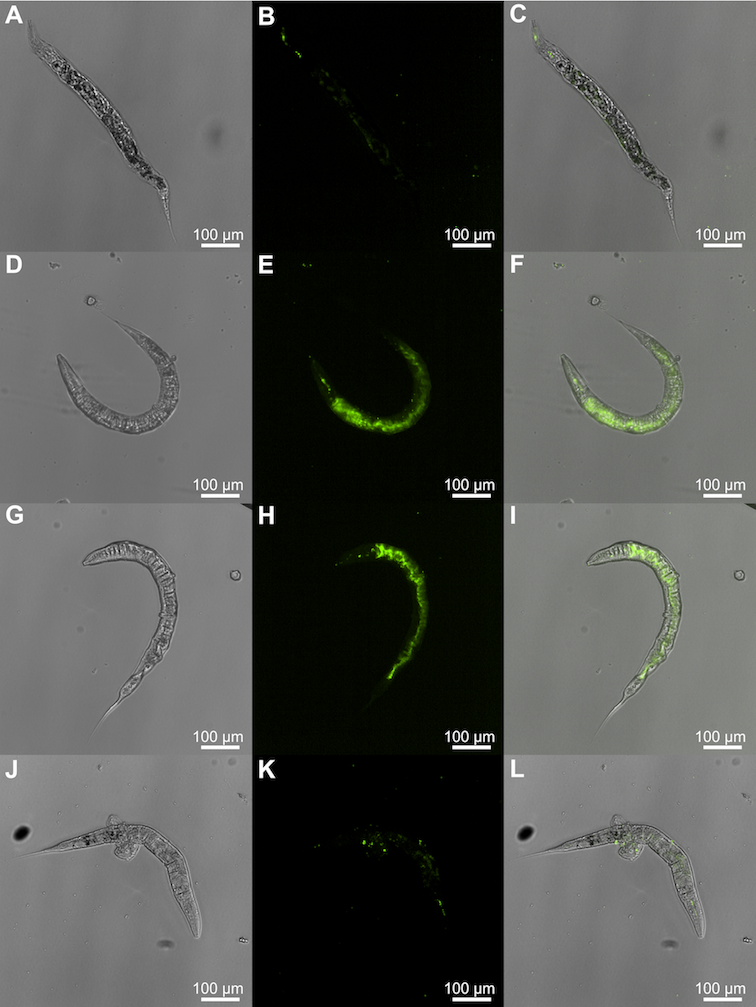

Supplement: FIG S3 [file sph003182553sf3.tif]

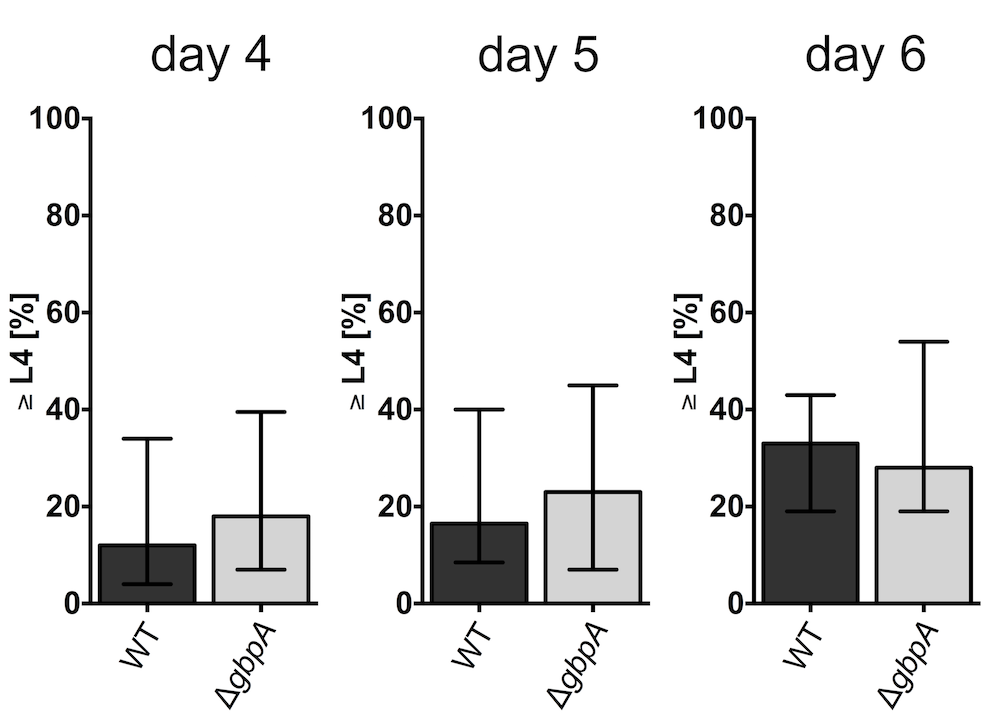

Supplement: FIG S4 [file sph003182553sf4.tif]
