# Supplementary material for: Genes Activated by Vibrio cholerae upon Exposure to Caenorhabditis elegans Reveal the Mannose-Sensitive Hemagglutinin To Be Essential for Colonization
Source: mSphere. 2018 May 23;3(3):e00238-18. doi: 10.1128/mSphereDirect.00238-18 (PMC5967197; doi:10.1128/mSphereDirect.00238-18)
Supplement: TABLE S2 [file sph003182553st2.docx]

**TABLE S2: Bacterial strains and plasmids used in the study.**

| **Strain/ Plasmid** | **Relevant genotype/ resistance** | **Source** |
| --- | --- | --- |
| ***E. coli* strains** |  |  |
| OP50 | *E. coli* | Caenorhabditis Genetics Center |
| DH5αλ*pir* | F^-^ ɸ80∆*lacZ*∆*M15*∆(*argF lac*)*U169 deoR recA1 endA1 hsdR17* (r_K_^-^m_K_^+^) *supE44 thi-1 gyrA69 relA1,* λ *pir*R6K, Ap^R^ | ([1](#_ENREF_47)) |
| SM10λ*pir* | *thi thr leu tonA lacY supE recA*::RPA-2-Te::Mu *λpir*R6K, Km^R^ | ([2](#_ENREF_48)) |
| ***V. cholerae* strains** |  |  |
| WT | *V. cholerae* AC53, spontaneous Sm^r^ mutant of *V. cholerae* E7946 (O1; biotype: El Tor; serotype: Ogawa; clinical isolate from Bahrain 1978) | ([3, 4](#_ENREF_49)) |
| AC53res | insertion of res*-neo-sacB-*res in *lacZ* of *V. cholerae* WT | (5) |
| AC53res1 | insertion of res1*-neo-sacB-*res1 in *lacZ* of *V. cholerae* WT | (5) |
| *∆mshA* | *V. cholerae* AC53 with deletion of gene VC0409 (*mshA*) | This study |
| *∆gbpA* | *V. cholerae* AC53 with deletion of gene VCA0811 (*gpbA*) | This study |
| ***C. elegans* strains** |  |  |
| SS104 | *C. elegans* *glp-4(bn2)I* | Caenorhabditis Genetics Center |
| Bristol N2 | *C. elegans* | Caenorhabditis Genetics Center |
| **Plasmids** |  |  |
| p | pMMB67EH, IncQ broad-host-range cloning vector, Ap^r^ | ([6](#_ENREF_51)) |
| pCVD442 | *oriR6K mobRP4 sacB*, Ap^r^ | ([7](#_ENREF_40)) |
| pIVET5nlibrary | library of random *V. cholerae* genomic fragments ligated into pGOA1193, pGOA1194 and pGOA1195 to create transcriptional fusions to *tnpR* | ([8](#_ENREF_52)) |
| pmshA | VC0409 in pMMB67EH, Ap^r^ | This study |
| pCVD442∆mshA | suicide vector for deletion of *mshA* | This study |
| pgfp | pAR181 with *gfp*mut3b | ([6](#_ENREF_51)) |

1. Hanahan D. 1983. Studies on transformation of Escherichia coli with plasmids. J Mol Biol 166:557-80.

2. Miller VL, Mekalanos JJ. 1988. A novel suicide vector and its use in construction of insertion mutations: osmoregulation of outer membrane proteins and virulence determinants in *Vibrio cholerae* requires *toxR*. J Bacteriol 170:2575-83.

3. Miller VL, DiRita VJ, Mekalanos JJ. 1989. Identification of toxS, a regulatory gene whose product enhances toxR-mediated activation of the cholera toxin promoter. J Bacteriol 171:1288-93.

4. Vance RE, Zhu J, Mekalanos JJ. 2003. A constitutively active variant of the quorum-sensing regulator LuxO affects protease production and biofilm formation in Vibrio cholerae. Infect Immun 71:2571-6.

5. Schild S, Tamayo R, Nelson EJ, Qadri F, Calderwood SB, Camilli A. 2007. Genes Induced Late in Infection Increase Fitness of Vibrio cholerae after Release into the Environment. Cell Host & Microbe 2:264-277.

6. Sherlock O, Schembri MA, Reisner A, Klemm P. 2004. Novel roles for the AIDA adhesin from diarrheagenic Escherichia coli: cell aggregation and biofilm formation. J Bacteriol 186:8058-65.

7. Donnenberg MS, Kaper JB. 1991. Construction of an eae deletion mutant of enteropathogenic Escherichia coli by using a positive-selection suicide vector. Infection and Immunity 59:4310-4317.

8. Osorio CG, Crawford JA, Michalski J, Martinez-Wilson H, Kaper JB, Camilli A. 2005. Second-Generation Recombination-Based In Vivo Expression Technology for Large-Scale Screening for Vibrio cholerae Genes Induced during Infection of the Mouse Small Intestine. Infection and Immunity 73:972-980.
