# Supplementary material for: Genes Activated by Vibrio cholerae upon Exposure to Caenorhabditis elegans Reveal the Mannose-Sensitive Hemagglutinin To Be Essential for Colonization
Source: mSphere. 2018 May 23;3(3):e00238-18. doi: 10.1128/mSphereDirect.00238-18 (PMC5967197; doi:10.1128/mSphereDirect.00238-18)
Supplement: TABLE S3 [file sph003182553st3.docx]

**TABLE 3: Oligonucleotides used in this study.**

| **Oligonucleotides** | **Sequence** ^a^ |
| --- | --- |
| VC0409-*Sac*I_1 | 5’-AATTCTAGATTCCAATGCAACATGGG-3’ |
| VC0409-*Nco*I_2 | 5’-AATCCATGGCATCTCTCTTTCATGTGAATACGC-3’ |
| VC0409-*Nco*I_3 | 5’-AATCCATGGTAATTTAAATATGGCTCGTGCA-3’ |
| VC0409-*Xba*I_4 | 5’-AATGAGCTCTTTACCTAACGCGGATG-3’ |
| VCA0811-*Sac*I_1 | 5’-TTTGAGCTCGTCAGGTCTTTGGTATT-3 |
| VCA0811-*Eco*RI_2 | 5’-TTTGAATTCCATCACAGACTCTTCTTTGTT-3 |
| VCA0811-*Eco*RI_3 | 5’-TTTGAATTCTAAGTTATCCTCCCTCTTACA-3 |
| VCA0811-*Xba*I_4 | 5’-TTTTCTAGAGACTTTCTCTGGATGGG-3 |
| VC0409-*Sac*I_fw | 5’-TTTGAGCTCGCGTATTCACATGAAAGAGAGATG-3’ |
| VC0409-*Xba*I_rv | 5’-TTTTCTAGAACATGCACGAGCCATATTTAAATTA-3’ |
| IVET-1 | 5’-AAATCGTACGCCGACTAGAATGTC-3’ ^b^ |
| IVET-2 | 5’-ACGTCACCTTCCTCCACCTTCATC-3’ ^b^ |
| IVET-3 | 5’-CCTTCATCCTCAGCAAGTCCA-3’ ^b^ |

^a^ restriction sites are underlined

^b^ oligonucleotides according to Schild et al. (Schild S, Tamayo R, Nelson EJ, Qadri F, Calderwood SB, Camilli A. 2007. Genes Induced Late in Infection Increase Fitness of *Vibrio cholerae* after Release into the Environment. Cell Host & Microbe 2:264-277.)
